# Persistent symptoms and risk factors predicting prolonged time to symptom-free after SARS‑CoV‑2 infection: an analysis of the baseline examination of the German COVIDOM/NAPKON-POP cohort

**DOI:** 10.1007/s15010-023-02043-6

**Published:** 2023-05-25

**Authors:** Yanyan Shi, Ralf Strobl, Christian Apfelbacher, Thomas Bahmer, Ramsia Geisler, Peter Heuschmann, Anna Schäfer, Hanno Hoven, Thomas Keil, Michael Krawczak, Lilian Krist, Christina Lemhöfer, Wolfgang Lieb, Bettina Lorenz-Depiereux, Rafael Mikolajczyk, Felipe A. Montellano, Jens Peter Reese, Stefan Schreiber, Nicole Skoetz, Stefan Störk, Jörg Janne Vehreschild, Martin Witzenrath, Eva Grill

**Affiliations:** 1 Institute for Medical Information Processing, Biometry and Epidemiology (IBE), Faculty of Medicine, Ludwig-Maximilians-Universität München (LMU Munich), Marchioninistr. 15, 81377 Munich, Germany; 2 Pettenkofer School of Public Health, Munich, Germany; 3 German Center for Vertigo and Balance Disorders, University Hospital, Ludwig-Maximilians-Universität München (LMU Munich), Munich, Germany; 4 Institute of Social Medicine and Health Systems Research, Medical Faculty, Otto von Guericke University Magdeburg, Magdeburg, Germany; 5 Internal Medicine Department I, University Hospital Schleswig-Holstein Campus Kiel (UKSH Kiel), Kiel, Germany; 6 Department II of Internal Medicine, Hematology/Oncology, Goethe University, Frankfurt, Frankfurt Am Main, Germany; 7 Institute for Clinical Epidemiology and Biometry, Julius-Maximilians-University, Würzburg, Würzburg, Germany; 8 Clinical Trial Center, University Hospital Würzburg, Würzburg, Germany; 9 Institute for Occupational and Maritime Medicine, University Medical Center Hamburg-Eppendorf, Hamburg, Germany; 10 Institute of Social Medicine, Epidemiology and Health Economics, Charité-Universitätsmedizin Berlin, Berlin, Germany; 11 State Institute of Health I, Bavarian Health and Food Safety Authority, Erlangen, Germany; 12 Institute of Medical Informatics and Statistics, Kiel University, University Medical Center Schleswig-Holstein, Kiel, Germany; 13 Institute of Physical and Rehabilitation Medicine, University Hospital Jena, Jena, Germany; 14 Institute of Epidemiology, Kiel University, University Medical Center Schleswig-Holstein, Kiel, Germany; 15 Research Unit Molecular Epidemiology, Institute of Epidemiology, Helmholtz Center Munich, Munich, Germany; 16 Institute for Medical Epidemiology, Biometrics, and Informatics, Interdisciplinary Center for Health Sciences, Medical Faculty of the Martin Luther University Halle-Wittenberg, Halle (Saale), Germany; 17 German Centre for Mental Health, Site Jena-Magdeburg-Halle, Halle, Germany; 18 Department of Neurology, University Hospital Würzburg, Würzburg, Germany; 19 Evidence-Based Medicine, Department I of Internal Medicine, Faculty of Medicine and University Hospital Cologne, University of Cologne, Cologne, Germany; 20 Department of Clinical Research and Epidemiology, Comprehensive Heart Failure Center and Department of Internal Medicine I, University Hospital Würzburg, Würzburg, Germany; 21 Department I of Internal Medicine, Faculty of Medicine and University Hospital Cologne, University of Cologne, Cologne, Germany; 22 German Centre for Infection Research (DZIF), Partner Site Bonn‑Cologne, Cologne, Germany; 23 Department of Infectious Diseases, Respiratory Medicine and Critical Care, Charité-Universitätsmedizin Berlin, Corporate Member of Freie Universität Berlin and Humboldt-Universität zu Berlin, Berlin, Germany; 24 German Center for Lung Research (DZL), Giessen, Germany

**Keywords:** COVID-19, Long COVID, Post-COVID syndrome, Time to symptom-free, Risk factors

## Abstract

**Purpose:**

We aimed to assess symptoms in patients after SARS-CoV-2 infection and to identify factors predicting prolonged time to symptom-free.

**Methods:**

COVIDOM/NAPKON-POP is a population-based prospective cohort of adults whose first on-site visits were scheduled ≥ 6 months after a positive SARS-CoV-2 PCR test. Retrospective data including self-reported symptoms and time to symptom-free were collected during the survey before a site visit. In the survival analyses, being symptom-free served as the event and time to be symptom-free as the time variable. Data were visualized with Kaplan–Meier curves, differences were tested with log-rank tests. A stratified Cox proportional hazard model was used to estimate adjusted hazard ratios (aHRs) of predictors, with aHR < 1 indicating a longer time to symptom-free.

**Results:**

Of 1175 symptomatic participants included in the present analysis, 636 (54.1%) reported persistent symptoms after 280 days (SD 68) post infection. 25% of participants were free from symptoms after 18 days [quartiles: 14, 21]. Factors associated with prolonged time to symptom-free were age 49–59 years compared to < 49 years (aHR 0.70, 95% CI 0.56–0.87), female sex (aHR 0.78, 95% CI 0.65–0.93), lower educational level (aHR 0.77, 95% CI 0.64–0.93), living with a partner (aHR 0.81, 95% CI 0.66–0.99), low resilience (aHR 0.65, 95% CI 0.47–0.90), steroid treatment (aHR 0.22, 95% CI 0.05–0.90) and no medication (aHR 0.74, 95% CI 0.62–0.89) during acute infection.

**Conclusion:**

In the studied population, COVID-19 symptoms had resolved in one-quarter of participants within 18 days, and in 34.5% within 28 days. Over half of the participants reported COVID-19-related symptoms 9 months after infection. Symptom persistence was predominantly determined by participant’s characteristics that are difficult to modify.

**Supplementary Information:**

The online version contains supplementary material available at https://doi.org/10.1007/s15010-023-02043-6.

## Introduction

As of December 2022, severe acute respiratory syndrome coronavirus 2 (SARS‑CoV‑2) infection has been confirmed in over 600 million people worldwide [1]. Many patients, even those with mild-to-moderate acute symptoms, continue to suffer from symptoms after acute disease [2, 3]. “Long COVID” is increasingly used as an umbrella term for signs and symptoms persisting for 4 weeks or longer after SARS-CoV-2 infection [4].

The most frequently reported persisting symptoms include fatigue, dyspnea, sleep disorders or insomnia, headache, attention disorders, anosmia and ageusia [5–10]. A systematic review of 151 studies revealed that > 50% of COVID-19 patients still had at least one symptom 12 months after a confirmed infection [11]. However, generalizability to the general population is hampered by the fact that many studies investigating persisting symptoms after SARS-CoV-2 infection were based on hospitalized patients whilst others drew upon small, selected samples, or lacked a sufficiently long follow-up period [12–16]. The ongoing German COVIDOM/NAPKON-POP population-based study included participants ≥ 6 months after a positive SARS-CoV-2 polymerase chain reaction (PCR) test, regardless of disease severity. Recently, some of us used the first results of this study [9] to develop a severity score to quantify the symptom load associated with post-COVID syndrome (PCS score), which is broadly synonymous with Long COVID. PCS score facilitates an objective assessment of the extent and severity of the condition in the general population. However, detailed information on the health burden of long COVID, specifically on the time to full recovery, remains scarce.

A study from the Netherlands reported a median time to complete recovery of 63 days among individuals with mild, and 232 days among individuals with moderate disease severity [17]. A large international online survey of patients with suspected and confirmed SARS-CoV-2 infection revealed that the probability of time to recovery from symptoms exceeding 35 weeks was 91.8% [18]. Most eminent risk factors for Long COVID were the presence or number of existing comorbidities [2, 17, 19], however, results on risks of individual comorbidities were inconsistent [13, 20–22]. Treatment during acute infection such as steroid or antibiotic medication was not indicative of a complete recovery [23]. Up to date, the time course of COVID-19 symptoms and factors associated with time to recovery are thus still incompletely understood.

Using COVIDOM/NAPKON-POP baseline data, we aimed to retrospectively assess the time course of symptom persistence after SARS-CoV-2 infection. We also investigated factors predicting prolonged time to complete recovery (i.e., to becoming symptom-free) in this multi-center population-based study covering three regions of Germany.

## Methods

### Study design

The National Pandemic Cohort Study Network (“Nationales Pandemie Kohorten Netz”, NAPKON) was established in Germany in 2020 to coordinate and harmonize COVID-19 research at a nation-wide level [24]. NAPKON-POP is the population-based platform that hosts the COVIDOM study aimed at investigating the long-term consequences of COVID-19. Participants in COVIDOM/NAPKON-POP were recruited at three study sites in Germany, namely Kiel, Würzburg, and the Neukölln district of Berlin, covering defined geographical regions in the vicinity.

### Participants

All eligible individuals were identified through the mandatory registration of a positive SARS-CoV-2 PCR test by local health authorities. First on-site visits of prospective participants were scheduled ≥ 6 months post PCR test, regardless of their acute disease severity, following procedures detailed elsewhere [25]. Inclusion criteria of participants were: (a) positive PCR for SARS-CoV-2 ≥ 6 months before enrollment, (b) living in one of the three covered regions, (c) ≥ 18 years of age, and (d) written informed consent. Exclusion criterion was an acute SARS-CoV-2 re-infection at the time of the initial questionnaire, or at the scheduled site visit [25]. Recruitment and follow-up of the COVIDOM/NAPKON-POP cohort are still ongoing. For the present analysis, data from participants recruited between November 2020 and September 2021 were used, and only symptomatic participants were included.

### Method of data collection

Retrospective data on the acute course of COVID-19, time to symptom-free and current symptoms were collected from self-filled questionnaires before the on-site visit. Later, participants were assessed at the study sites during enrollment into the prospective cohort study, collecting data on body measurement, resilience, COVID-19 treatment, comorbidities, and lifestyles by physical examination, questionnaires, and interviews [25].

### Measures

#### Symptoms

**Fig. 1:**
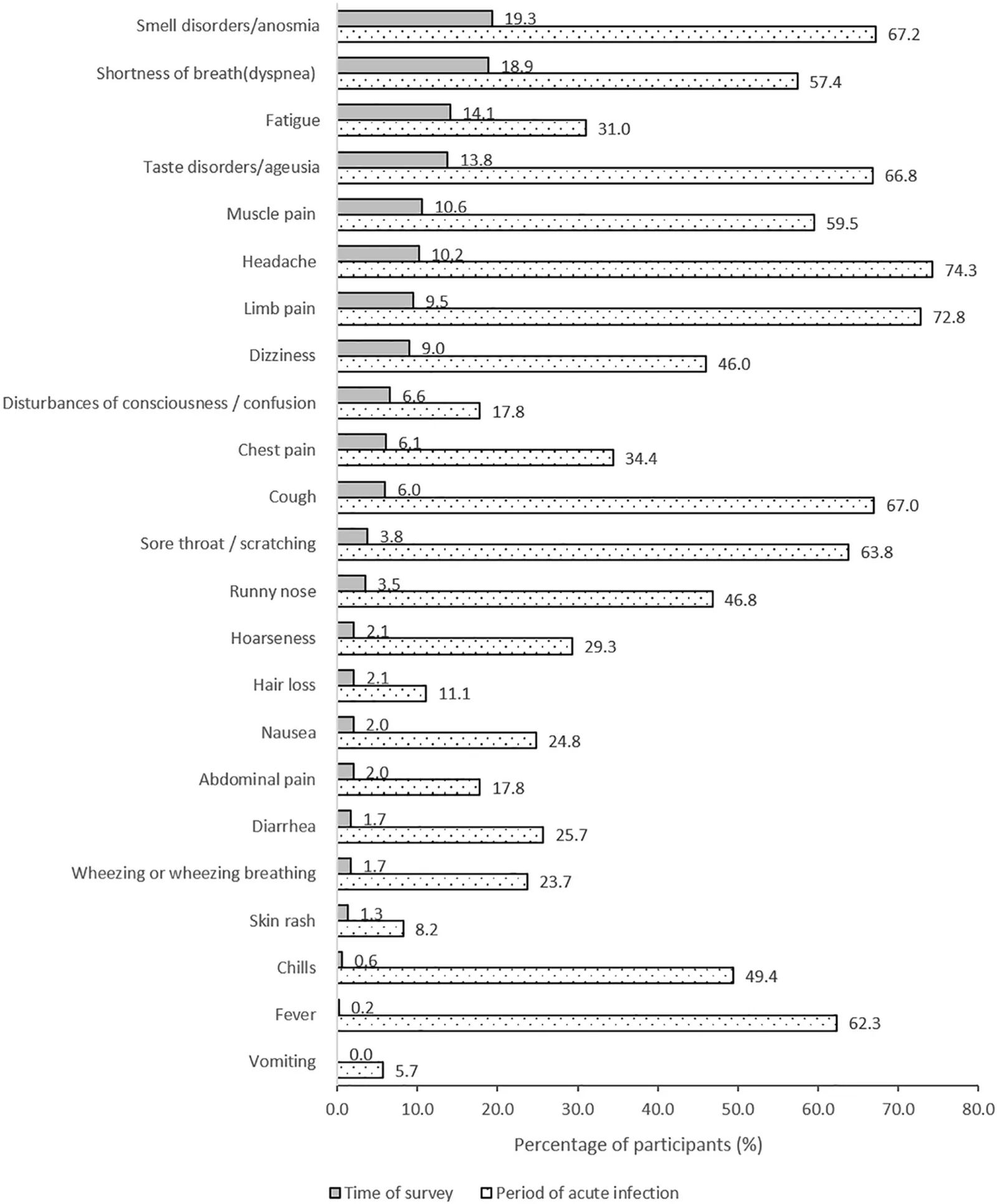
COVID-19 related symptoms during acute infection and time of survey (*N* = 1175)

COVID-19-related symptoms were assessed by a self-selection from 22 specific symptoms and “other symptoms” [9]. Participants were asked whether they experienced these symptoms in either the infection/acute period or at the time of the survey (“current symptoms”). Fatigue was considered present when the free-text answer to the prompting question following “other symptoms” contained “fatigue” or its synonyms. A list of all 23 symptoms is provided in Fig. 1. Presence of current symptoms was assessed by the question “Do you still have symptoms currently?”.

#### Time to symptom-free

Time to symptom-free was assessed using the question: “How long did it take you to become symptom-free after the occurrence of first symptoms?” Time to symptom-free was measured as the time from the first appearance of symptoms to symptom-free status in days, weeks or months, re-scaled to days (7 days per week and 30 days per month) for the purpose of the present study.

For those still experiencing symptoms at the time of the survey, time to be symptom-free was considered as censored and was calculated as the time between the appearance of the first symptoms and the survey.

Additionally, we tested for group differences up to 28 days (i.e. before becoming a Long COVID case) by manually censoring data at this time point. In detail, we set the symptom-free time to 28 days and the symptom status to “experiencing symptoms” whenever getting symptom-free took longer than 28 days.

#### Alcohol consumption

Alcohol consumption was categorized as abstainers, low-risk alcohol consumption, or risky alcohol consumption (i.e. ≥ 5 times per week, or consumption on one occasion ≥ 4 or ≥ 5 glasses for women and men, respectively) [26].

#### Body Mass Index (BMI)

BMI was calculated from the weight and height measurements taken at the study site with the formula BMI = kg/m^2^ and was categorized as: underweight (BMI < 18.5), normal (18.5 ≤ BMI < 25), pre-obese (25 ≤ BMI < 30), or obese (BMI ≥ 30) [27].

#### Resilience

Resilience was measured by the 6-item Brief Resilience Scale and was categorized as: low (1.00–2.99), normal (3.00–4.30), and high (4.31–5.00). The Brief Resilience Scale can be found in Supplementary Appendix (S Table 1).

#### COVID-19 treatment

COVID-19 treatment was assessed by the question: “Have you taken any medications for SARS-Cov-2 infection?” together with prompting three treatment categories of steroids, anticoagulation, and anti-infectives. In the present analysis, we merged corticosteroids, steroids (> 0.5 mg/kg prednisone equivalents) and steroids (≤ 0.5 mg/kg prednisone equivalents) into one variable “steroids”.

#### Comorbidities

Comorbidities were self-reported physician-diagnosed diseases. (Detailed in Table 1).

**Table 1:** Characteristics of the final sample and asymptomatic participants

Characteristics	*n* (%)		*P* value
	Symptomatic participants (*n* = 1175)	Asymptomatic participants (*n* = 108)	
*Age (years)*			< 0.001*
< 49	589 (50.1)	48 (44.4)	
49–59	346 (29.4)	27 (25.0)	
≥ 60	236 (20.1)	26 (24.1)	
Missings	4 (0.3)	7 (6.5)	
*Sex*			0.0538
Female	659 (56.1)	48 (44.4)	
Male	515 (43.8)	60 (55.6)	
Missings	1 (0.1)	0 (0.0)	
*Nationality*			< 0.001*
German	1143 (97.3)	63 (58.3)	
Non-German	29 (2.5)	4 (3.7)	
Missings	3 (0.3)	41 (38.0)	
*Educational level*			< 0.001*
University entrance certificate	665 (56.6)	33 (30.6)	
Lower education	498 (42.4)	32 (29.6)	
Missings	12 (1.0)	43 (39.8)	
*Living status*			< 0.001*
Living with a partner	820 (69.8)	46 (42.6)	
No partner/not living with a partner	287 (24.4)	19 (17.6)	
Missings	68 (5.8)	43 (39.8)	
*Smoking status*			< 0.001*
Current-smokers	143 (12.2)	11 (10.2)	
Ex-smokers	436 (37.1)	18 (16.7)	
Non-smokers	587 (50.0)	32 (29.6)	
Missings	9 (0.8)	47 (43.5)	
*Alcohol consumption*			0.4274
Abstainer	101 (8.6)	12 (11.1)	
Low-risk alcohol consumption	605 (51.5)	49 (45.4)	
Risky alcohol consumption	147 (12.5)	18 (16.7)	
Missings	322 (27.4)	29 (26.9)	
*Hospitalization during acute infection*			0.8953
Hospitalized	75 (6.4)	6 (5.6)	
Non-hospitalized	1100 (93.6)	102 (94.4)	
*Symptom burden during acute infection*			< 0.001*
No symptom	0 (0.0)	108 (100.0)	
1–5 symptoms	200 (17.0)	0 (0.0)	
≥ 6 symptoms	975 (83.0)	0 (0.0)	
*Body mass index*			0.7529
Normal	465 (39.6)	38 (35.2)	
Obese	282 (24.0)	29 (26.9)	
Pre-obese	416 (35.4)	41 (38.0)	
Underweight	10 (0.9)	0 (0.0)	
Missings	2 (0.2)	0 (0.0)	
*Resilience*			0.0523
Low resilience	212 (18.0)	14 (13.0)	
Normal resilience	690 (58.7)	58 (53.7)	
High resilience	163 (13.9)	18 (16.7)	
Missings	110 (9.4)	18 (16.7)	
*COVID-19 treatment*			
Treated with medication	641 (54.6)	29 (26.9)	< 0.001*
Antipyretics	540 (46.0)	24 (22.2)	< 0.001*
Missings	17 (1.4)	3 (2.8)	
Steroids	20 (1.7)	0 (0.0)	0.2738
Missings	13 (1.1)	2 (1.9)	
Anticoagulation	64 (5.4)	3 (2.8)	0.3199
Missings	13 (1.1)	2 (1.9)	
Anti-infectives	49 (4.2)	3 (2.8)	0.6167
*Comorbidities*			
Number of comorbidities			0.5658
0	403 (34.3)	40 (37.0)	
1	364 (31.0)	36 (33.3)	
≥ 2	408 (34.7)	32 (29.6)	
Chronic liver disease	116 (9.9)	11 (10.2)	0.3305
Missings	117 (10.0)	6 (5.6)	
Chronic rheumatologic/immunologic disease	104 (8.9)	7 (6.5)	0.6454
Missings	16 (1.4)	2 (1.9)	
Tumor/cancer disease	21 (1.8)	2 (1.9)	1.0000
Missings	4 (0.3)	0 (0.0)	
Chronic neurological disease	307 (26.1)	23 (21.3)	0.1306
Missings	12 (1.0)	3 (2.8)	
Lung disease	226 (19.2)	16 (14.8)	0.0165*
Missings	13 (1.1)	5 (4.6)	
Ear, nose and throat disease	290 (24.7)	23 (21.3)	0.1650
Missings	24 (2.0)	5 (4.6)	
Cardiovascular disease	346 (29.4)	30 (27.8)	0.0368*
Missings	14 (1.2)	5 (4.6)	
Diabetes	46 (3.9)	5 (4.6)	< 0.001*
Missings	5 (0.4)	47 (43.5)	
*Current symptoms*			
Symptom-free	539 (45.9)		
Persistent symptoms	636 (54.1)		

*P* value: Pearson *χ*^2^ test (or Fisher exact test if expected *n* < 5)

*P < 0.05

### Statistical analysis

Mean, with standard deviation (SD), or median with quartiles were used for the description of continuous variables. Counts and percentages were used for the description of categorical variables.

In the survival analysis, being symptom-free served as the event and time to be symptom-free as the time variable. Since < 50% of symptomatic participants were symptom-free at the time of investigation, we reported the Q1 (25%) time to symptom-free, instead of the median time. Kaplan–Meier estimator served to estimate the survival function and Kaplan–Meier plots served to visualize the survival curves. Log-rank tests were used to test group differences in both overall survival curves and in survival curves up to 28 days.

Missing data were imputed by Multiple Imputation by Chained Equations (MICE) [28], yielding ten imputed datasets. Imputation was based on age, sex, educational level, living status, smoking, alcohol consumption, symptom burden during acute infection, BMI, COVID-19 treatment during acute infection, chronic liver disease, chronic rheumatologic/immunologic disease, tumor/cancer disease, chronic neurological disease, lung disease, ear, nose and throat (ENT) disease, cardiovascular disease, and diabetes. The final model was combined with Rubin’s rules, calculating final coefficient as the mean of coefficients estimated from imputed datasets and calculating the variance of estimated coefficients by factoring in the within and between imputation variance [29].

We applied a stratified Cox proportional hazard regression model to explore the factors predicting prolonged time to symptom-free after infection. Proportional hazard (PH) assumption was assessed with the Schoenfeld test [30]. Predictors violating the PH assumption were included as a stratified parameter in the multivariable Cox model [30]. By including a variable as a stratified parameter, the stratified Cox proportional hazard model sets a different baseline hazard corresponding to each stratum as defined by the variable, and then estimates common coefficients for the remaining explanatory variables except for the stratified variable, thus providing hazard ratios controlled for the effect of the stratification variable, but not for the stratification variable itself [30]. Symptom burden and hospitalization both violated the PH assumption and both are closely related to unmeasured disease severity during the acute infection phase. Since only 75 (6.4%) of all patients were hospitalized, we decided to only include symptom burden as a stratification parameter and analyzed the effect of hospitalization in a separate sensitivity analysis (see below). A Generalized Variance Inflation Factor (GVIF) was used to check for multicollinearity among covariates, GVIF^1/(2*Df)^ of ≥ 5 was considered indicative of collinearity [31]. Stepwise variable selection was conducted, selecting the model with the smallest Akaike information criterion. To assess the linearity assumption, we plotted the Martingale residuals against covariates. The adjusted hazard ratios (aHRs) were used to describe the hazard of becoming symptom-free, with aHR < 1 indicating a longer time to symptom free. A multivariate Wald test was used to assess the overall significance of difference for categorical variables with more than three categories. The concordance index (*C*-index) was used to measure the goodness-of-fit of the fitted models with ten imputed datasets; it measures the agreement between observed survival and predicted survival, with a value of 0.5 representing a random prediction and a value of 1.0 representing the best possible model prediction [32].

The threshold for statistical significance was set to 0.05. Since this was an exploratory study, no correction for multiple testing was applied. We used R (*version 4.1.1*) with the *dplyr, survival, car, MASS*, and *mice* packages for all statistical analyses. MS Office and R were used to create figures.

### Sensitivity analyses

To evaluate the robustness of the final model, we conducted separate Cox proportional hazard models for each potential risk factor adjusted for age and sex. To investigate the effect of hospitalization on time to symptom-free we conducted three separate models: the first model only for patients having been hospitalized during acute infection, the second model for patients not having been hospitalized, and the third model including hospitalization with two different effect estimates, one for the effect in the first four weeks and one afterwards.

## Results

### Study participants

**Fig. 2:**
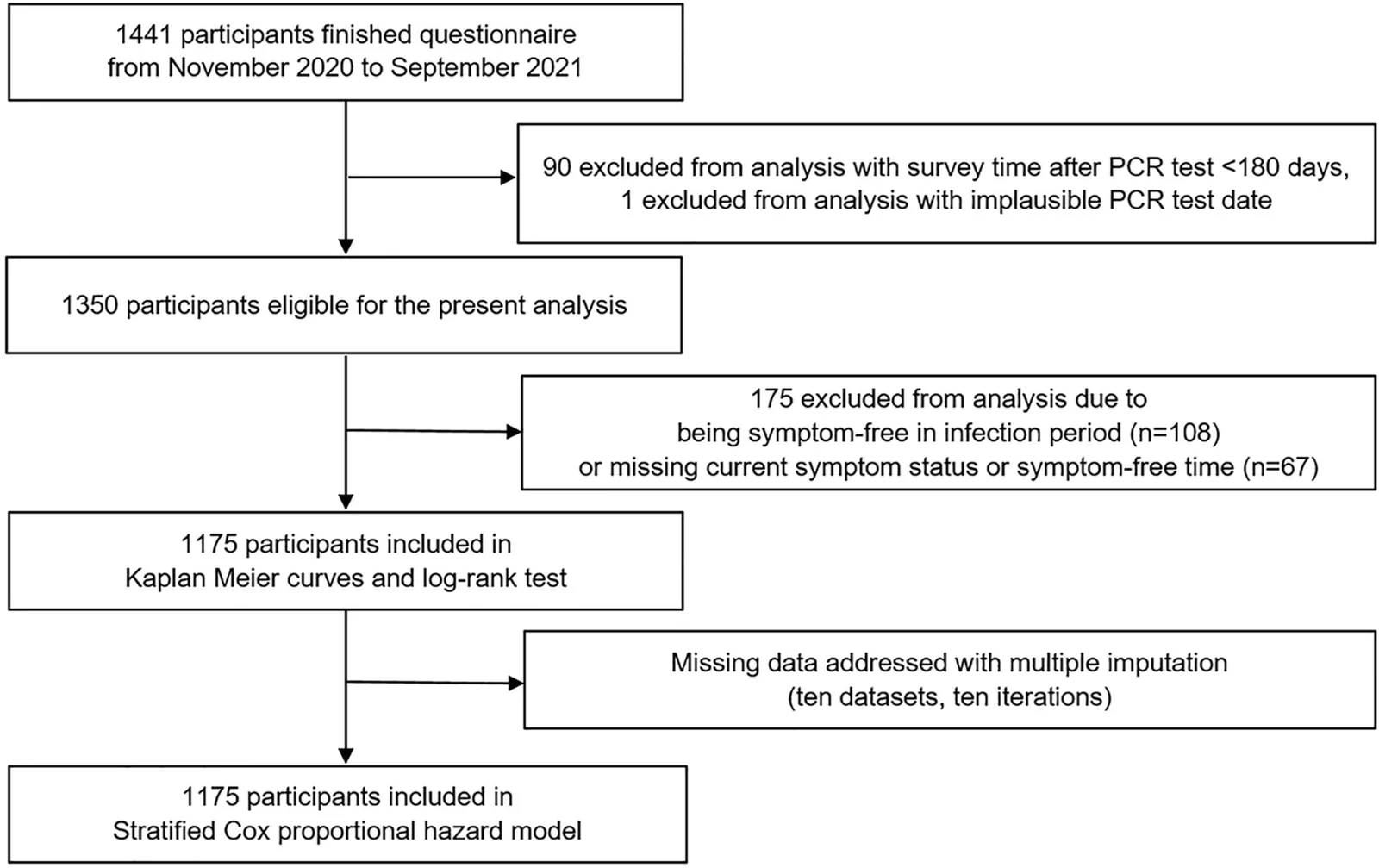
Study profile

Data from 1441 COVIDOM/NAPKON-POP participants were available, including 1126 from Kiel, 208 from Würzburg, and 107 from Berlin. After excluding 90 cases with a time between PCR test and survey of < 6 months, and one case with an implausible PCR test date, 1350 participants were eligible for the present analysis. Of these, 108 participants had been asymptomatic during the acute phase, information on the current symptom status or the time to symptom-free of another 67 participants were missing. They were thus excluded from the analyses, resulting in a final sample of 1175 participants (Fig. 2).

Mean time since the onset of infection for 1175 participants was 280 days (SD 68). 54.1% of initially symptomatic participants continued to experience symptoms. Sex, BMI, resilience and most comorbidities of symptomatic participants were comparable to asymptomatic participants, whereas age, nationality, educational level, living status, smoking status, and COVID-19 treatment were not (Table 1).

### Persistent COVID-19-related symptoms

At the time of survey, 22 of 23 different symptoms from the acute phase were still persistent: anosmia (19.3%), dyspnea (18.9%), fatigue (14.1%), and ageusia (13.8%) were the most common persisting symptoms. Muscle pain, headache, limb pain, dizziness, disturbances of consciousness/confusion, chest pain, and cough were reported by > 5% of participants each. Over 40% of participants had suffered from sore throat, fever, chills, and a runny nose during acute infection, while only < 5% reported these symptoms at the time of the survey, respectively (Fig. 1).

### Time to symptom-free

**Fig. 3:**
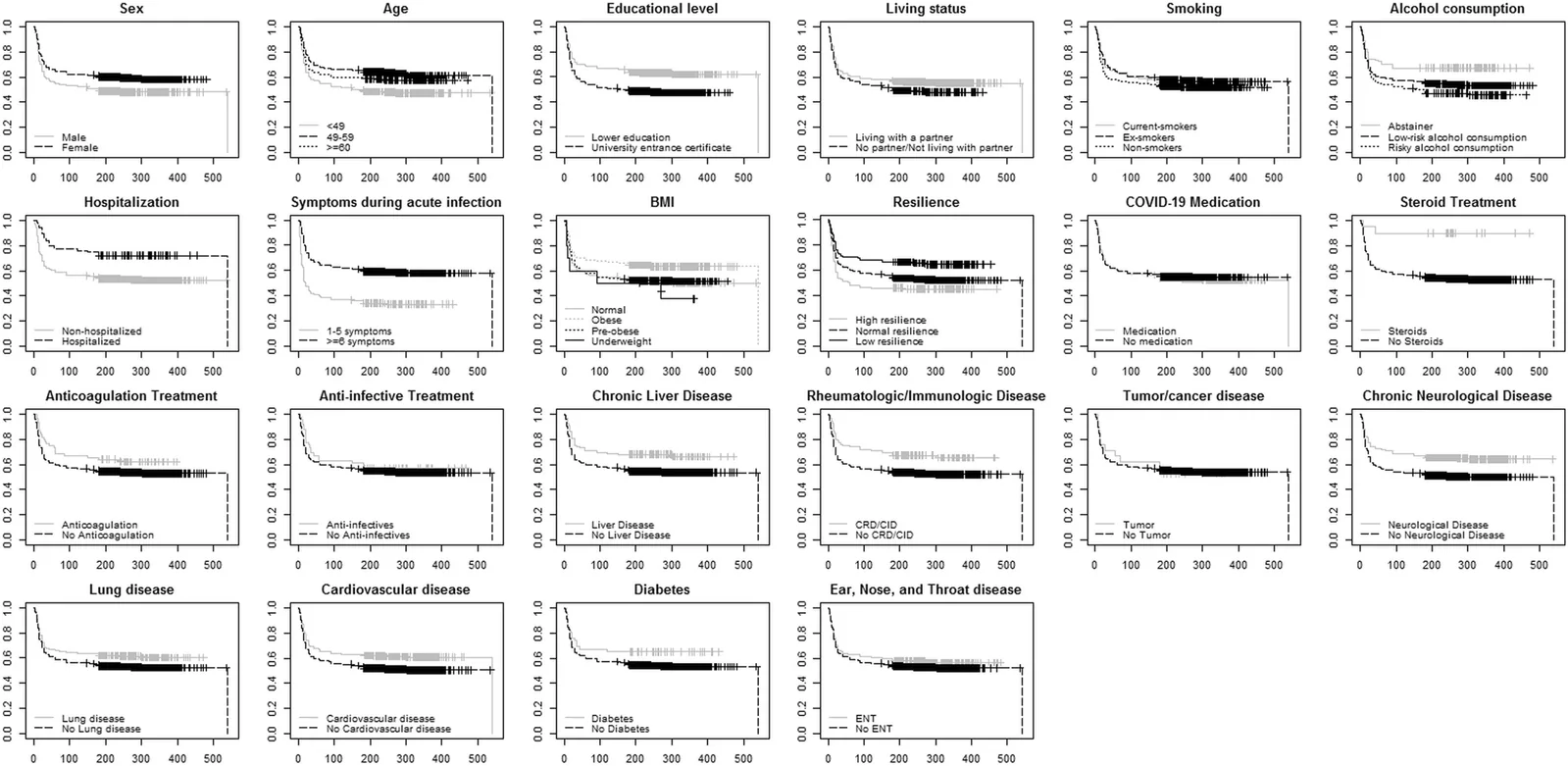
Survival curves of time to symptom-free status for different patient groups (*N* = 1175). *X*-axis is the time to symptom-free in days, *y*-axis is the percentage of participants not reaching a symptom-free status. *CRD/CID:* chronic rheumatologic/immunologic disease

Figure 3 and Table 2 summarize the observed bivariate differences in symptom persistence. Q1 time to symptom-free was 18 days [quartiles: 14 days, 21 days]. 405 (34.5%) participants had become symptom-free during the first 28 days since symptom onset, and only slow symptom resolution was seen afterwards. Time to symptom-free differed according to age, sex, educational level, living status, alcohol consumption, hospitalization during acute infection, symptom burden during acute infection, BMI, resilience, steroid treatment during acute infection, chronic liver disease, chronic rheumatologic/immunologic disease, chronic neurological disease, lung disease, and cardiovascular disease. Similar results were obtained when testing for group differences in survival curves up to 28 days, except for living status, smoking status, alcohol consumption, BMI, anticoagulation treatment and lung disease.

**Table 2:** Time to symptom-free status in patients stratified by patient characteristics (*N* = 1175)

Characteristics	Q1 time to symptom-free status	95% confidence interval	% of symptom-free patients 9 months after infection	Difference in survival curves**	
				Whole observation time	First 28 days
*Age*					
< 49	14	[14; 15]	52.3	< 0.001*	< 0.001*
49–59	28	[21; 42]	37.6		
≥ 60	20	[14; 28]	42.4		
*Sex*					
Female	21	[18; 28]	41.1	< 0.001*	0.0010*
Male	14	[14; 18]	51.8		
*Educational level*					
University entrance certificate	14	[14; 28]	52.0	< 0.001*	0.0003*
Lower education	21	[21; 35]	37.3		
*Living status*					
Living with a partner	21	[14; 21]	44.5	0.0295*	0.0972
No partner/Not living with a partner	14	[14; 20]	51.6		
*Smoking status*					
Current-smokers	17	[14; 42]	45.5	0.1584	0.0082*
Ex-smokers	21	[20; 28]	43.3		
Non-smokers	14	[14; 18]	48.0		
*Alcohol consumption*					
Abstainer	21	[18; NA]	32.7	0.0102*	0.0946
Low-risk alcohol consumption	17	[14; 21]	46.3		
Risky alcohol consumption	14	[14; 21]	53.7		
*Hospitalization during acute infection*					
Hospitalized	150	[42; NA]	29.3	< 0.001*	< 0.001*
Non-hospitalized	14	[14; 21]	47.0		
*Symptom burden during acute infection*					
1–5 symptoms	7	[6; 10]	66.5	< 0.001*	< 0.001*
≥ 6 symptoms	21	[21; 28]	41.6		
*BMI*					
Normal	14	[14; 21]	49.0	0.0037*	0.0648
Obese	21	[18; 60]	36.5		
Pre-obese	19	[14; 21]	48.1		
Underweight	10	[7; NA]	60.0		
*Resilience*					
Low resilience	38	[21; 90]	34.4	< 0.001*	< 0.001*
Normal resilience	17	[14; 21]	47.4		
High resilience	14	[10; 18]	54.6		
*Treated with medication*					
Yes	20	[14; 21]	46.6	0.8998	0.6708
No	14	[14; 21]	44.9		
*Steroids*					
Yes	NA	[NA; NA]	10.0	0.0040*	0.0107*
No	17	[14; 21]	46.6		
*Anticoagulation*					
Yes	49	[21; NA]	37.5	0.1005	0.0145*
No	17	[14; 21]	46.4		
*Anti-infectives*					
Yes	30	[21; 180]	42.9	0.4359	0.1079
No	17	[14; 21]	46.0		
*Chronic liver disease*					
Yes	32.5	[21; NA]	32.8	0.0055*	0.0113*
No	14	[14; 21]	46.0		
*Chronic rheumatologic/immunologic disease*					
Yes	51	[21; NA]	33.7	0.0051*	0.0026*
No	14	[14; 21]	47.3		
*Tumor/cancer diseases*					
Yes	28	[10; NA]	47.6	0.9996	0.5793
No	18	[14; 21]	45.8		
*Chronic neurological disease*					
Yes	28	[21; 90]	35.2	< 0.001*	< 0.001*
No	14	[14; 20]	49.5		
*Lung disease*					
Yes	21	[18; 28]	38.9	0.0332*	0.2364
No	14	[14; 21]	47.3		
*ENT disease*					
Yes	21	[14; 28]	43.1	0.2100	0.4942
No	17	[14; 21]	47.4		
*Cardiovascular disease*					
Yes	21	[21; 28]	39.0	0.0019*	0.0323*
No	14	[14; 20]	48.7		
*Diabetes*					
Yes	30	[14; NA]	34.8	0.1553	0.1516
No	18	[14; 21]	46.3		

Q1: first quartile; number of days until 25% of participants became symptom-free

*P < 0.05

**P-values were the result of the respective log-rank tests

### Prognostic analyses

Symptom burden during acute infection was included as a stratification variable in the final model because it violated the PH assumption. All GVIF were smaller than 5. Other variables included in the final model were age, sex, educational level, living status, alcohol consumption, BMI, resilience, COVID-19 medication and steroid treatment during acute infection, chronic liver disease, chronic rheumatologic/immunologic disease, and chronic neurological disease. The concordance indices of the ten fitted models ranged between 0.6305 and 0.6401.

Patients aged 49–59 years had a 30% lower hazard of becoming symptom-free than those aged < 49 years (aHR 0.70, 95% CI 0.56–0.87), while the hazard for patients ≥ 60 years did not differ from that < 49 years. Prolonged time to recovery was also seen in women (aHR 0.78, 95% CI 0.65–0.93), and patients with lower educational level (aHR 0.77, 95% CI 0.64–0.93), or living with a partner (aHR 0.81, 95% CI 0.66–0.99), or with low resilience (aHR 0.65, 95% CI 0.47–0.90). Steroid treatment (aHR 0.22, 95% CI 0.05–0.90) and no medication (aHR 0.74, 95% CI 0.62–0.89) during acute infection also increased time to symptom-free (Table 3).

**Table 3:** Risk factors predicting prolonged time to symptom-free status in COVID-19 patients stratified by symptom burden during acute infection (*N* = 1175, stratified Cox proportional hazard model)

Covariates	Adjusted hazard ratio	95% confidence interval	*P* value	Overall *P* value
*Age*				
< 49	Reference			0.0053*
49–59	0.70	[0.56; 0.87]	0.0013*	
≥ 60	0.92	[0.72; 1.17]	0.4857	
*Sex*				
Male	Reference			NA
Female	0.78	[0.65; 0.93]	0.0073*	
*Educational level*				
University entrance certificate	Reference			NA
Lower education	0.77	[0.64; 0.93]	0.0062*	
*Living status*				
No partner/not living with a partner	Reference			NA
Living with a partner	0.81	[0.66; 0.99]	0.0382*	
*Alcohol consumption*				
Abstainer	Reference			0.1851
Low-risk alcohol consumption	1.31	[0.94; 1.81]	0.1102	
Risky alcohol consumption	1.41	[0.98; 2.04]	0.0687	
*Body Mass Index*				
Normal	Reference			0.1596
Underweight	1.40	[0.61; 3.17]	0.4259	
Pre-obese	1.04	[0.85; 1.27]	0.7237	
Obese	0.80	[0.63; 1.03]	0.0826	
*Resilience*				
High resilience	Reference			0.0327*
Normal resilience	0.83	[0.65; 1.05]	0.1281	
Low resilience	0.65	[0.47; 0.90]	0.0090*	
*Treated with medication*				
Yes	Reference			NA
No	0.74	[0.62; 0.89]	0.0013*	
*Steroid treatment*				
No	Reference			NA
Yes	0.22	[0.05; 0.90]	0.0357*	
*Chronic liver disease*				
No	Reference			NA
Yes	0.81	[0.58; 1.15]	0.2385	
*Chronic rheumatologic/immunologic disease*				
No	Reference			NA
Yes	0.71	[0.50; 1.00]	0.0512	
*Chronic neurological disease*				
No	Reference			NA
Yes	0.80	[0.64; 1.00]	0.0522	

Overall *P* value: multivariate Wald test

*P < 0.05

Age and sex-adjusted coefficients for each potential risk factor can be found in the Supplementary Appendix (S Table 2). Cox proportional hazard models for hospitalized patients and non-hospitalized patients, together with time-varying effect estimates of hospitalization can be found in the Supplementary Appendix (S Table 3–5). Non-hospitalized patients were more likely to become symptom-free in the first four weeks (aHR 2.42, 95% CI 1.28–4.59). No significant differences were found after this time period.

## Discussion

### Main findings

We used data from a large population-based multicenter study for the retrospective analysis of the duration of, and risk factors for a prolonged recovery from acute SARS-CoV-2 infection. While 65.5% of included participants reported to still have symptoms 28 days after infection, over half of the symptomatic participants (54.1%) experienced at least one persisting symptom about 9 months post-infection. 22 of 23 different symptoms during the acute phase except for vomiting persisted beyond 9 months, with anosmia, dyspnea, ageusia, and fatigue being the most frequent ones. We found that female sex, age between 49 and 59 years, lower educational level, living with a partner, low resilience, steroid treatment and no medication during acute infection were associated with prolonged time to symptom-free, and being hospitalized was associated with prolonged time only in the first four weeks.

### Study findings in context

We found that COVID-19-related symptoms rapidly resolved at the beginning but only incremental improvement was seen beyond 28 days. A former study also demonstrated that symptom load at 1.5 to 6 months was not associated with the length of time since symptom onset, suggesting that improvement in symptoms primarily occurred during the first few weeks after infection [12]. Furthermore, most subgroup differences in time to symptom-free occurred within 28 days after symptom onset in our study.

The most prevalent symptoms including anosmia, dyspnea, ageusia, and fatigue corresponded to those reported in a study of non-hospitalized individuals and another one of patients with mild or moderate symptoms [12, 16]. Long persistence of symptoms is worrying because persisting COVID-19 symptoms are associated with poor health-related quality of life (HRQOL) [9, 33]. Even though the present analysis did not differentiate symptoms according to their severity or their impact on daily life or HRQOL, our previous analysis of COVIDOM/NAPKON-POP data [9] revealed that different symptoms have a different impact on the severity of PCS and, consequently, on HRQOL. Therefore, learning more about symptom persistence and symptom resolution is of utmost clinical relevance.

Our study identified several risk factors for prolonged symptom persistence. An age between 49 and 59 years, being female, lower education, living with a partner, low resilience, steroid treatment, and no medication during acute infection were factors that predicted longer symptom persistence. Some of these factors like age are in line with previous studies [21, 34], although the inverse U-shaped association of age with risk might seem surprising. However, similar results were obtained from 10 longitudinal studies in the UK, with the highest risk noted in the middle age categories, i.e. 45–54 and 55–69 years [20]. Arguably, this might be attributable to competing mortality risks or erroneous attribution of symptoms to other causes in older age [20]. On the other hand, we cannot exclude that participants’ differential recall might also have been determined by some of the risk factors in question, especially age, resilience, and education. Hence, the identified predictors still require confirmation by independent longitudinal studies. Consistent with most previous studies [21, 23, 35, 36], we found that female patients were less likely to recover quickly from symptoms than male patients. In contrast to our results, a Swedish study found that the female sex was protective for Long COVID-related sick leave, but only in a subgroup of hospitalized patients [37]. Patients with lower education are more likely to have physically demanding jobs [38], which might have influenced their recovery from symptoms. The effect of living status might be due to recall bias since patients living with a partner might have discussed their symptoms more frequently with their partner, as compared to patients without a partner or not living with a partner. This might result in differential reporting of symptoms in patients without a partner or not living with a partner, thus the observed effect should be interpreted with caution*.* Moreover, it may be speculated that constant exposure to a partner’s infection might have increased virus load. In our previous study [9], we found low resilience and strong acute disease severity to be risk factors for severe PCS. Similarly, patients with more severe acute COVID-19 were also reported to show prolonged symptoms [39]. Likewise, steroid treatment might be an indicator of disease severity that results in prolonged symptoms. Although it has been shown that inhaled corticosteroid treatment improved symptom resolution in COVID-19 patients [40], a meta-analysis demonstrated an association between corticosteroid therapy and increased length of stay, although this finding was only based on subgroup analysis in three randomized controlled trials [41].

### Strengths and limitations

A major strength of our study is that we reported a population-based estimate of the status and duration of symptoms drawing upon data from over 1100 COVID-19 patients with an average follow-up of 9 months.

There are some limitations. First and foremost, our use of the COVIDOM/NAPKON-POP time-to-recovery data had to be retrospective in nature because the study did not collect symptoms prospectively starting from infection. Since this might have been subject to recall bias, factors affecting the precision of the derived time-to-recovery data might have confounded some of the relationships between the latter and potential predictors. However, it is also likely that patients remember the time course well even after recovery. Second, as this study is not a representative sample of the total population, selection bias must be taken into account. It has to be mentioned that selection and differential response could have biased the estimates of the prevalence and persistence of symptoms. However, given the nature of the cooperation with the local health authorities, we are confident that the COVIDOM/NAPKON-POP sample is a valid representation of the infected population at the given time in the respective regions. Third, symptom status was collected by self-report, asking participants about COVID-19-related symptoms. However, we cannot rule out the possibility that some symptoms were caused by other respiratory infections. Furthermore, although we assume that most participants would not mention a chronic symptom as it is not noticeably related to the COVID-19 disease, future studies should evaluate the presence of symptoms before COVID-19 and their potential aggravation because of COVID-19. Fourth, long-term symptom status of initially asymptomatic patients was not evaluated. It is still unknown whether this group developed new symptoms after acute infection. Third, patients included in COVIDOM/NAPKON study probably mainly had SARS-CoV-2 wild type or alpha variant infection with a higher burden of symptoms than later variants. Future analyses of the cohort population from 2022 will evaluate how comparable symptom persistence after the omicron variant is to our present findings. Finally, the study does not include a control group, which makes it difficult to know whether the reported symptoms can indeed be attributed to SARS-CoV-2 infection.

### Conclusions

Over half of the participants reported COVID-19-related symptoms 9 months after infection. Many patients experienced rapid recovery, but prolonged recovery was also seen particularly among those characterized by middle age, female sex, lower educational level, living with a partner, low resilience, and without medication during acute infection.

## Supplementary Information

Below is the link to the electronic supplementary material.

Supplementary file1 (DOCX 31 KB)

## Data Availability

Data of this study are available upon request to the Use & Access Committee (UAC) of NAPKON (https://proskive.napkon.de).
