# Supplementary material for: Persistent symptoms and risk factors predicting prolonged time to symptom-free after SARS‑CoV‑2 infection: an analysis of the baseline examination of the German COVIDOM/NAPKON-POP cohort
Source: Infection. 2023 May 25;51(6):1679–94. doi: 10.1007/s15010-023-02043-6 (PMC10212223; doi:10.1007/s15010-023-02043-6)
Supplement: Supplementary file 1 — Supplementary file1 (DOCX 31 KB) [file 15010_2023_2043_MOESM1_ESM.docx]

**Persistent symptoms and risk factors predicting prolonged time to symptom-free after SARS‑CoV‑2 infection: An analysis of the baseline examination of the German COVIDOM/NAPKON-POP** **cohort**

Yanyan Shi, Ralf Strobl, Christian Apfelbacher, Thomas Bahmer, Ramsia Geisler, Peter Heuschmann, Anna Horn, Hanno Hoven, Thomas Keil, Michael Krawczak, Lilian Krist, Christina Lemhöfer, Wolfgang Lieb, Bettina Lorenz-Depiereux, Rafael Mikolajczyk, Felipe A. Montellano, Jens Peter Reese, Stefan Schreiber, Nicole Skoetz, Stefan Störk, Jörg Janne Vehreschild, Martin Witzenrath, Eva Grill  *on behalf of the NAPKON Study Group*

**Corresponding author:**

Prof. Dr. Eva Grill, Institute for Medical Information Processing, Biometry and Epidemiology, Ludwig-Maximilians-Universität München, Marchioninistr. 15, 81377 Munich, Germany. **E-Mail:** Eva.Grill@med.uni-muenchen.de

**SUPPLEMENTARY DATA**

**S Table1 Brief Resilience Score**

| **Item** |
| --- |
| 1. I tend to bounce back quickly after hard times. |
| 2. I have a hard time making it through stressful events. (R) |
| 3. It does not take me long to recover from a stressful event. |
| 4. It is hard for me to snap back when something bad happens. (R) |
| 5. I usually come through difficult times with little trouble. |
| 6. I tend to take a long time to get over set-backs in my life. (R) |

R = reverse coded items

Response options: 1 = strongly disagree, 2 = disagree, 3 = neutral, 4 = agree, 5 = strongly agree.

**S Table 2 Risk factors predicting prolonged time to symptom-free status in COVID-19 patients (N=1175, separate Cox proportional hazard models for each risk factor adjusted for age and sex)**

| **Covariates** | **Adjusted hazard ratio** | **95% confidence interval** | **P value** |
| --- | --- | --- | --- |
| **Educational level** |  |  |  |
| University entrance certificate | Reference |  |  |
| Lower education | 0.70 | [0.58; 0.84] | 0.0002* |
| **Living status** |  |  |  |
| No partner/Not living with a partner | Reference |  |  |
| Living with a partner | 0.85 | [0.69; 1.03] | 0.1024 |
| **Smoking status** |  |  |  |
| Current-smokers | Reference |  |  |
| Ex-smokers | 1.04 | [0.78; 1.39] | 0.7860 |
| Non-smokers | 1.24 | [0.95; 1.63] | 0.1205 |
| **Alcohol consumption** |  |  |  |
| Abstainer | Reference |  |  |
| Low-risk alcohol consumption | 1.40 | [1.00; 1.96] | 0.0493* |
| Risky alcohol consumption | 1.58 | [1.08; 2.30] | 0.0186* |
| **Body Mass Index** |  |  |  |
| Normal | Reference |  |  |
| Underweight | 1.22 | [0.54; 2.74] | 0.6347 |
| Pre-obese | 0.97 | [0.80; 1.18] | 0.7674 |
| Obese | 0.69 | [0.54; 0.87] | 0.0019* |
| **Resilience** |  |  |  |
| High resilience | Reference |  |  |
| Normal resilience | 0.77 | [0.61; 0.98] | 0.0342* |
| Low resilience | 0.56 | [0.42; 0.76] | 0.0003* |
| **Treated with medication** |  |  |  |
| Yes | Reference |  |  |
| No | 0.92 | [0.78; 1.10] | 0.3579 |
| **Steroid treatment** |  |  |  |
| No | Reference |  |  |
| Yes | 0.20 | [0.05; 0.82] | 0.0253* |
| **Anticoagulation** |  |  |  |
| No | Reference |  |  |
| Yes | 0.74 | [0.49; 1.12] | 0.1591 |
| **Anti-infectives** |  |  |  |
| No | Reference |  |  |
| Yes | 0.94 | [0.60; 1.45] | 0.7717 |
| **Chronic liver disease** |  |  |  |
| No | Reference |  |  |
| Yes | 0.67 | [0.48; 0.94] | 0.0199* |
| **Chronic rheumatologic/immunologic disease** |  |  |  |
| No | Reference |  |  |
| Yes | 0.68 | [0.48; 0.96] | 0.0297* |
| **Tumor/cancer diseases** |  |  |  |
| No | Reference |  |  |
| Yes | 1.16 | [0.62; 2.18] | 0.6512 |
| **Chronic neurological disease** |  |  |  |
| No | Reference |  |  |
| Yes | 0.69 | [0.56; 0.85] | 0.0006* |
| **Lung disease** |  |  |  |
| No | Reference |  |  |
| Yes | 0.80 | [0.63; 1.01] | 0.0587 |
| **ENT disease** |  |  |  |
| No | Reference |  |  |
| Yes | 0.94 | [0.77; 1.15] | 0.5403 |
| **Cardiovascular disease** |  |  |  |
| No | Reference |  |  |
| Yes | 0.80 | [0.64; 1.00] | 0.0468* |
| **Diabetes** |  |  |  |
| No | Reference |  |  |
| Yes | 0.77 | [0.47; 1.27] | 0.2998 |

Note: Age and sex adjusted coefficients for symptom burden during acute infection and hospitalization during acute infection were not presented due to violation of proportional hazard assumption.

* P < 0.05.

**S Table 3 Risk factors predicting prolonged time to symptom-free status in hospitalized COVID-19 patients (N=75, Cox proportional hazard model)**

| **Covariates** | **Adjusted hazard ratio** | **95% confidence interval** | **P value** |
| --- | --- | --- | --- |
| **Alcohol consumption** |  |  |  |
| Abstainer | Reference |  |  |
| Low-risk alcohol consumption | 2.90 | [0.63; 13.30] | 0.1944 |
| Risky alcohol consumption | 3.76 | [0.71; 19.86] | 0.1429 |
| **Symptom burden during acute infection** |  |  |  |
| 1-5 symptoms | Reference |  |  |
| ≥6 symptoms | 0.09 | [0.03; 0.32] | 0.0020* |
| **Treated with medication** |  |  |  |
| Yes | Reference |  |  |
| No | 0.27 | [0.08; 0.88] | 0.0459* |
| **Chronic rheumatologic/immunologic disease** |  |  |  |
| No | Reference |  |  |
| Yes | 0.32 | [0.07; 1.48] | 0.1656 |

* P < 0.05.

**S Table 4 Risk factors predicting prolonged time to symptom-free status in non-hospitalized COVID-19 patients stratified by symptom burden during acute infection (N=1100, stratified Cox proportional hazard model)**

| **Covariates** | **Adjusted hazard ratio** | **95% confidence interval** | **P value** |
| --- | --- | --- | --- |
| **Age** |  |  |  |
| <49 | Reference |  |  |
| 49-59 | 0.71 | [0.57; 0.88] | 0.0022* |
| ≥60 | 1.00 | [0.77; 1.28] | 0.9821 |
| **Sex** |  |  |  |
| Male | Reference |  |  |
| Female | 0.78 | [0.65; 0.94] | 0.0078* |
| **Educational level** |  |  |  |
| University entrance certificate | Reference |  |  |
| Lower education | 0.74 | [0.61; 0.89] | 0.0018* |
| **Living status** |  |  |  |
| No partner/Not living with a partner | Reference |  |  |
| Living with a partner | 0.82 | [0.66; 1.00] | 0.0512 |
| **Alcohol consumption** |  |  |  |
| Abstainer | Reference |  |  |
| Low-risk alcohol consumption | 1.26 | [0.85; 1.86] | 0.2501 |
| Risky alcohol consumption | 1.35 | [0.89; 2.04] | 0.1569 |
| **Resilience** |  |  |  |
| High resilience | Reference |  |  |
| Normal resilience | 0.86 | [0.68; 1.10] | 0.2229 |
| Low resilience | 0.64 | [0.46; 0.89] | 0.0089* |
| **Treated with medication** |  |  |  |
| Yes | Reference |  |  |
| No | 0.76 | [0.63; 0.92] | 0.0039* |
| **Steroid treatment** |  |  |  |
| No | Reference |  |  |
| Yes | 0.20 | [0.03; 1.43] | 0.1094 |
| **Chronic liver disease** |  |  |  |
| No | Reference |  |  |
| Yes | 0.79 | [0.55; 1.11] | 0.1755 |
| **Chronic rheumatologic/immunologic disease** |  |  |  |
| No | Reference |  |  |
| Yes | 0.71 | [0.50; 1.02] | 0.0661 |
| **Chronic neurological disease** |  |  |  |
| No | Reference |  |  |
| Yes | 0.80 | [0.64; 1.01] | 0.0594 |

* P < 0.05.

**S Table 5 Effect of hospitalization on COVID-19 patients stratified by symptom burden during acute infection (N=1175, stratified Cox proportional hazard model). The model is adjusted for age, sex, educational level, living status, alcohol consumption, BMI, resilience, COVID-19 medication, steroid treatment, chronic liver disease, chronic rheumatologic/immunologic disease, and chronic neurological disease.**

| **Covariates** | **Adjusted hazard ratio** | **95% confidence interval** | **P value** |
| --- | --- | --- | --- |
| **Hospitalization during acute infection** |  |  |  |
| Hospitalized | Reference |  |  |
| Non-hospitalized : first four weeks | 2.42 | [1.28; 4.59] | 0.007* |
| Non-hospitalized : after four weeks | 0.79 | [0.42; 1.48] | 0.457 |

* P < 0.05.
